# Insights into the mechanisms of apoptosis and pathogenesis in enterovirus 71 infections: A review

**DOI:** 10.1097/MD.0000000000042183

**Published:** 2025-04-11

**Authors:** Jia-Mei Wu, Cheng-Si Wang, Xi-Wen Yu

**Affiliations:** a School of Basic Medicine, Baicheng Medical College, Baicheng, China; b College of Mathematical Sciences, Shanghai Jiaotong University, Shanghai, China; c Department of Acupuncture and Moxibustion, Baicheng Medical College, Baicheng, China.

**Keywords:** advance, apoptosis, enterovirus 71, pathogenesis, programmed cell death

## Abstract

This study examines the intricate interactions between enterovirus 71 (EV71) and various programmed cell death pathways, specifically apoptosis, necroptosis, and pyroptosis, which collectively shape the pathogenesis and severity of EV71 infections. Primarily affecting children under 5 years of age, EV71 is a leading cause of hand, foot, and mouth disease and has been linked to severe neurological and systemic complications. This paper highlights how EV71 leverages distinct cell death mechanisms to enhance viral replication and amplify disease pathology. Apoptosis, for example, may restrict viral dissemination by systematically eliminating infected cells; however, EV71’s activation of necroptosis and pyroptosis induces robust inflammatory responses, potentially resulting in extensive tissue damage and adverse health outcomes. Additionally, this study also summarizes recent advancements in the field, with an emphasis on experimental studies and clinical trials focused on vaccine and antiviral therapy development. Despite substantial progress, challenges persist, notably in achieving reliable vaccine efficacy and formulating safe treatment options specifically for pediatric populations. Moving forward, the review suggests that future research should delve further into understanding EV71-related complications, developing broad-spectrum antiviral agents, and investigating host genetic factors that may influence disease progression and outcomes. Ultimately, this research is essential for the development of targeted interventions capable of reducing severe symptoms without compromising the immune response, underscoring the importance of these efforts for public health and the management of infectious diseases.

## 1. Introduction

Enterovirus 71 (EV71), a non-enveloped, single-stranded RNA virus of the Picornaviridae family, has been under close scientific scrutiny since its first isolation in California in 1969.^[1,2]^ The virus primarily transmits through fecal-oral routes, respiratory secretions, or direct contact, often thriving in unsanitary conditions.^[2]^ Predominantly affecting children, especially those under 5, EV71 is a major causative agent of hand, foot, and mouth disease.^[3]^ While most infections manifest as mild to moderate symptoms such as fever, rash, and oral ulcers, EV71 can also lead to severe complications including encephalitis, aseptic meningitis, acute flaccid paralysis, and in rare instances, cardiopulmonary failure, which can be life-threatening.^[3]^ Consequently, studying this virus is crucial for public health.

The pathogenesis of EV71 involves complex roles of programmed cell death (PCD) mechanisms.^[4]^ PCD, an intrinsic cellular process of regulated self-destruction, plays a vital role in maintaining homeostasis and responding to infections within organisms. During EV71 infection, the virus activates various PCD pathways, including apoptosis, necroptosis, and pyroptosis.^[5,6]^ These different forms of cell death not only contribute to the elimination of the virus but are also implicated in the tissue damage and pathological processes induced by the virus. For instance, apoptosis of neuronal cells during EV71 infection is closely associated with the neurological complications that arise.

Given the significance of PCD in the context of EV71 infection, this review extensively explores how EV71 manipulates host cell death mechanisms to regulate its replication and spread, and the impact of these mechanisms on the outcomes of the infection. By delving into the different PCD modalities induced by EV71 and their molecular mechanisms, we aim to enhance the understanding of the pathological characteristics of EV71 and provide a scientific basis for developing preventive and therapeutic strategies against severe EV71 infections.

## 2. Basic concepts of PCD

Programmed cell dealth is a fundamental biological mechanism essential for maintaining cellular homeostasis and the proper functioning of multicellular organisms.^[7]^ This process enables cells to self-terminate in a regulated and orderly fashion. PCD encompasses several distinct types, notably apoptosis, necroptosis, and pyroptosis, each characterized by unique triggers and molecular pathways.

Apoptosis is widely recognized as a key form of PCD, often referred to as “cellular suicide.” It involves a series of biochemical events leading to specific morphological changes such as cell shrinkage, nuclear fragmentation, chromatin condensation, and DNA fragmentation. Apoptosis is regulated by an array of proteins, including the caspases, which are cysteine-aspartic proteases crucial for the execution phase of cell death.^[8,9]^ Additionally, the Bcl-2 family of proteins plays a pivotal role in this process by either promoting or inhibiting apoptosis.^[9]^ This form of cell death is vital for eliminating damaged or unnecessary cells, thereby preventing potential malignancies and aiding in tissue remodeling and repair.

Necroptosis represents a form of programmed necrosis or inflammatory cell death.^[10]^ Contrasting with apoptosis, necroptosis leads to cell rupture, releasing cellular contents that can provoke inflammation in adjacent tissues.^[10]^ This cell death pathway is mediated by receptor-interacting protein kinases 1 and 3 (RIPK1, RIPK3) and the mixed lineage kinase domain-like pseudokinase (MLKL), which collectively form the necrosome.^[11]^ Necroptosis is generally activated when apoptotic pathways are blocked, serving as an alternative mechanism to control cellular proliferation and as a defense strategy against pathogens by disrupting their replication in host cells.^[11]^

Pyroptosis is an inflammatory form of PCD often induced by microbial infections that involves the activation of gasdermin proteins, which form pores in the cell membrane.^[12]^ This leads to cell swelling, rupture, and ultimately, death. Pyroptosis plays a critical role in the immune defense by eliminating the replication niches for pathogens and by promoting the release of cytokines and other inflammatory signals, thus enhancing the host’s immune response.^[13]^

Each type of PCD serves distinct physiological and pathological roles, acting as a critical control mechanism for cell populations and responding to various cellular stresses.^[14]^ A deeper understanding of the molecular mechanisms underlying these processes offers valuable insights into numerous diseases, including viral infections and cancer, and is crucial for developing targeted therapies that can manipulate these cell death pathways to treat or prevent diseases.

## 3. EV71 virus and PCD

**Figure 1.**
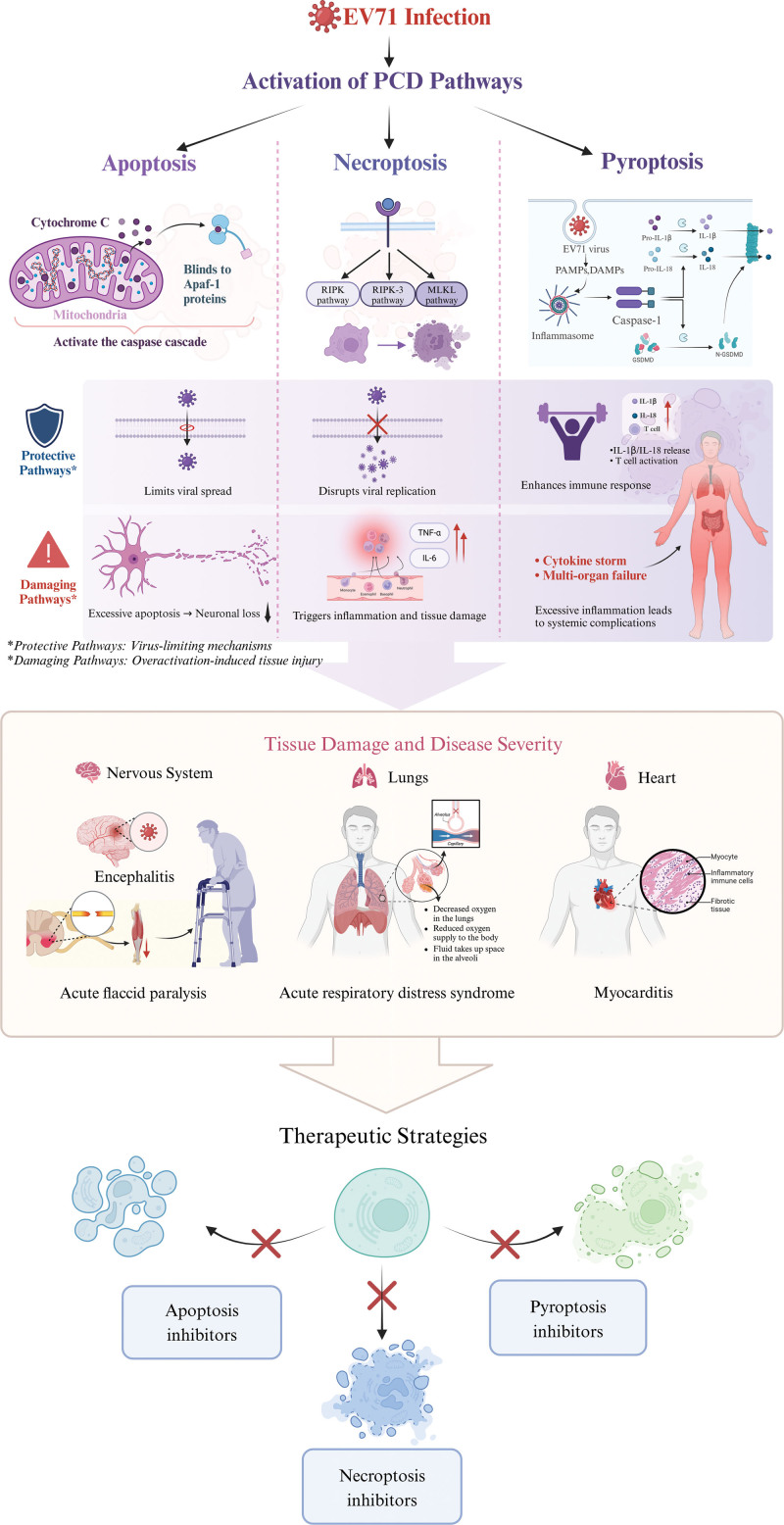
Interplay between EV71 infection and PCD. EV71 = enterovirus 71, PCD = programmed cell death.

Enterovirus 71 interacts intricately with PCD pathways, leveraging these mechanisms to facilitate its own life cycle while contributing to cellular damage and disease pathology.^[15]^ This review details how EV71 induces various forms of PCD, specifically apoptosis, necroptosis, and pyroptosis, and discusses their roles and interactions during infection (Fig. 1). Understanding these dynamics is vital for developing interventions that can mitigate severe outcomes without compromising viral clearance.

Apoptosis is frequently initiated upon EV71 infection. The virus engages mitochondrial pathways, disrupting mitochondrial integrity, which leads to the release of cytochrome c into the cytosol.^[16]^ This event triggers the activation of caspases, key proteins in the execution phase of apoptosis.^[17]^ By inducing apoptosis, EV71 can curtail the spread of the virus by prematurely killing infected cells, thus preventing full viral replication. However, excessive apoptosis, particularly in neuronal cells, is linked to the neurological symptoms observed in severe EV71 infections, indicating a detrimental aspect of this cell death pathway in the context of disease severity.

Necroptosis, unlike apoptosis, involves cellular lysis that releases inflammatory mediators, exacerbating tissue damage.^[18,19]^ EV71 can initiate necroptosis when apoptotic pathways are inhibited, via interactions that activate the RIPK1, RIPK3, and MLKL signaling cascade.^[20]^ This pathway culminates in the formation of the necrosome, leading to cell membrane rupture and inflammatory response. Necroptosis serves as a secondary mechanism of cell death, potentially beneficial in limiting viral replication by destroying the cellular machinery needed for virus production, yet it also contributes to inflammation and systemic damage.^[18,19]^

Pyroptosis is triggered by EV71 primarily in immune cells like macrophages, characterized by the activation of gasdermin proteins that form membrane pores, resulting in cell swelling and lysis.^[21,22]^ While pyroptosis effectively recruits immune responses to the infection site, enhancing pathogen clearance, it also leads to intense inflammation.^[22]^ In the case of EV71, this intense inflammatory response can result in systemic symptoms and, at times, severe clinical manifestations.

These interactions between EV71 and various PCD pathways reveal a sophisticated viral strategy: modulating cell death to balance between enhancing viral dissemination and evoking host immune responses.^[23]^ This manipulation can result in severe tissue damage and clinical symptoms, particularly when regulatory mechanisms fail to maintain a balance. Advanced understanding of these interactions is crucial for developing therapeutic strategies that aim to reduce the pathology associated with EV71 infections while preserving essential viral clearance mechanisms.

## 4. Association between PCD and the pathological mechanisms of EV71 virus

The interplay between PCD and the pathogenic mechanisms of EV71 profoundly affects both the disease’s progression and the host’s immune response.^[24]^ This relationship is pivotal for understanding the severe symptoms associated with EV71, such as neurological disorders, pulmonary distress, and cardiac issues, as well as for crafting targeted therapeutic strategies (Fig. 1).

Programmed cell death, which includes apoptosis, necroptosis, and pyroptosis, serves dual roles during EV71 infection.^[25]^ These cell death processes can restrict viral propagation by eliminating infected cells and curtailing virus production.^[25]^ However, unregulated or excessive activation of these pathways can cause significant tissue damage and escalate disease severity.^[26]^ Apoptosis, while typically containing viral infections and minimizing damage, can, if excessive, particularly within neural tissues, lead to cell loss and exacerbate neurological symptoms like acute flaccid paralysis and encephalitis.^[27]^ This suggests that although apoptosis controls viral spread, its excessive activation can be harmful, particularly in sensitive tissues.

Necroptosis, triggered when apoptosis pathways are blocked, releases cellular contents upon cell lysis, fueling inflammation and tissue damage.^[18]^ This form of cell death is linked to increased tissue damage in EV71 infections, particularly in the lungs and heart, where uncontrolled inflammation can lead to severe respiratory and cardiovascular symptoms.^[28]^ Pyroptosis, meanwhile, enhances inflammatory responses by releasing cytokines and chemokines, which attract immune cells to infection sites.^[29]^ While beneficial for clearing the virus, an excessive inflammatory response can lead to systemic complications, including shock and multi-organ failure.

The specific organ damage observed in severe EV71 cases can be directly linked to the activation of these PCD pathways.^[24]^ Apoptosis in neuronal cells contributes directly to neurodegeneration and the clinical manifestations of neurological impairments. Necroptosis in lung and heart tissues exacerbates inflammation, potentially leading to acute respiratory distress syndrome and myocarditis, respectively.^[30]^

From a therapeutic perspective, understanding the role of PCD in EV71 pathogenesis highlights potential interventions aimed at modulating these cell death pathways.^[31]^ The use of inhibitors of apoptosis proteins could prevent excessive apoptosis in neuronal tissues, potentially diminishing neurological symptoms.^[32]^ Necroptosis inhibitors, targeting components like RIPK1, RIPK3, or MLKL, might reduce tissue damage and inflammation in the lungs and heart.^[33]^ Additionally, cytokine blockers that mitigate the effects of pyroptosis could decrease severe inflammatory responses and improve overall outcomes in systemic infections.^[33]^

In summary, the development of therapeutic strategies that modulate PCD pathways could significantly enhance treatment outcomes for EV71 infections. Such strategies require a nuanced approach that balances the prevention of excessive cell death and uncontrolled inflammation while maintaining effective viral clearance.

## 5. Research advance and future directions

Research on EV71 has made considerable progress over recent years, enhancing our understanding of its epidemiology, pathogenesis, and interaction with host cellular mechanisms.^[1]^ This extensive research includes both experimental studies and clinical trials aimed at comprehensively understanding and effectively mitigating the impacts of EV71 infections.

Current research advance: The experimental studies have deepened our knowledge of the molecular biology of EV71, detailing how the virus invades host cells, replicates, and triggers immune responses.^[1]^ Key viral proteins and their interactions with host cell components have been identified, providing insights into the mechanisms of infection and replication.^[34]^ On the clinical front, efforts have concentrated on developing vaccines and antiviral therapies.^[35]^ Several vaccine candidates have been tested, showing promising results in preventing the severe manifestations of EV71 infections, particularly in regions where the virus is endemic.^[35]^

Current limitations and unresolved issues: Despite these advancements, several significant challenges remain. The efficacy of vaccines varies across different populations and age groups, potentially influenced by genetic factors or existing immunity levels. There is also a notable gap in effective, child-safe antiviral treatments for severe forms of the disease. Additionally, the mechanisms behind EV71-induced neurological damage are not fully understood, posing a hurdle to the development of targeted therapies aimed at preventing or mitigating these severe outcomes.

Future research directions: Looking forward, research should focus on several critical areas. Enhancing our understanding of how EV71 causes severe neurological, pulmonary, and cardiac complications is paramount. This could lead to the identification of novel therapeutic targets for preventing or treating severe disease manifestations. Developing broad-spectrum antiviral drugs that are effective against EV71 and other enteroviruses could significantly reduce the public health impact of these viruses. Further advancements in vaccine research are also needed to improve immunogenicity and efficacy across all demographics, including efforts towards universal vaccines that provide protection against multiple strains. Additionally, investigating the role of host genetic factors in disease susceptibility and severity may lead to personalized prevention and treatment strategies, allowing for interventions tailored to individual risk profiles.

By addressing these areas, the research community can continue to make significant strides towards controlling and potentially eradicating the threats posed by EV71, especially in vulnerable groups such as young children.

## 6. Summary

In summary, extensive research on the PCD pathways activated by EV71 has uncovered complex mechanisms by which the virus manipulates host cell fate, enhancing its own replication while contributing to disease progression. Studies have shown that EV71 selectively triggers apoptosis, necroptosis, and pyroptosis, balancing these pathways to both support viral spread and intensify the clinical severity of infection. Apoptosis serves as a containment mechanism by eliminating infected cells, thereby limiting viral dissemination. In contrast, the virus’s activation of necroptosis and pyroptosis induces robust inflammatory responses that can lead to significant tissue damage and increase the likelihood of severe health complications.

This mechanistic understanding is critical for public health, providing valuable insights that can inform both the management and prevention of EV71 infections. These findings lay a foundation for developing targeted therapeutic approaches designed to alleviate severe disease manifestations without impairing the immune system’s natural antiviral defenses. The potential clinical applications are substantial, offering strategies to reduce both morbidity and mortality associated with EV71, particularly in high-risk populations such as young children. Continued research into the influence of EV71 on PCD pathways is essential for advancing therapeutic options against this challenging virus, highlighting the importance of this work in the broader context of infectious disease management and public health policy.

## Acknowledgments

This study was partly supported by Natural Science Foundation of Jilin Province (YDZJ202301ZYTS197), Science and technology research project of the Education Department of Jilin Province (JJKH20231000KJ), Project of Baicheng Indicative Technology Development (No: 2023027), and The Project of Research and development of Baicheng licorice (BCGC202416). However, the supporter did not involve any work in this study.

## Author contributions

**Conceptualization:** Jia-Mei Wu, Cheng-Si Wang, Xi-Wen Yu.

**Data curation:** Jia-Mei Wu, Cheng-Si Wang, Xi-Wen Yu.

**Funding acquisition:** Jia-Mei Wu.

**Investigation:** Xi-Wen Yu.

**Methodology:** Cheng-Si Wang.

**Project administration:** Xi-Wen Yu.

**Resources:** Jia-Mei Wu, Cheng-Si Wang.

**Supervision:** Xi-Wen Yu.

**Validation:** Jia-Mei Wu, Cheng-Si Wang, Xi-Wen Yu.

**Visualization:** Jia-Mei Wu, Cheng-Si Wang, Xi-Wen Yu.

**Writing – original draft:** Jia-Mei Wu, Cheng-Si Wang, Xi-Wen Yu.

**Writing – review & editing:** Jia-Mei Wu, Cheng-Si Wang, Xi-Wen Yu.
